# Machine Learning–Predictive Models for Survival in Uterine Cancer Patients With Type 2 Diabetes: A Territory‐Wide Cohort Study

**DOI:** 10.1111/jog.70087

**Published:** 2025-09-30

**Authors:** Claire Chenwen Zhong, Junjie Huang, Zehuan Yang, Zhaojun Li, Yu Jiang, Jinqiu Yuan, Xiaodan Huang, Xiaofang Liu, Queran Lin, Han Wang, Jonathan Poon, Qi Dou, Irene Xin Yin Wu, Martin C. S. Wong

**Affiliations:** ^1^ The Jockey Club School of Public Health and Primary Care, Faculty of Medicine The Chinese University of Hong Kong Hong Kong SAR China; ^2^ Centre for Health Education and Health Promotion, Faculty of Medicine The Chinese University of Hong Kong Hong Kong SAR China; ^3^ Clinical Research Center & Big Data Center The Seventh Affiliated Hospital, Sun Yat‐Sen University Guangzhou Guangdong China; ^4^ Department of Radiation Oncology; State Key Laboratory of Oncology in South China; Guangdong Provincial Clinical Research Center for Cancer Sun Yat‐Sen University Cancer Center Guangzhou China; ^5^ Institute of Robotics and Automatic Information Systems, College of Artificial Intelligence Nankai University Tianjin China; ^6^ Guangdong Provincial Key Laboratory of Malignant Tumor Epigenetics and Gene Regulation, Breast Tumor Center, Clinical Research Design Division, Clinical Research Center Sun Yat‐Sen Memorial Hospital, Sun Yat‐Sen University Guangzhou China; ^7^ WHO Collaborating Centre for Public Health Education and Training, School of Public Health, Department of Primary Care and Public Health, Faculty of Medicine Imperial College London London UK; ^8^ Department of Gastroenterology, Tongji Hospital, Tongji Medical College Huazhong University of Science and Technology Wuhan Hubei China; ^9^ Information Technology and Health Informatics Division, Hospital Authority Hong Kong SAR China; ^10^ Department of Computer Science and Engineering, Faculty of Engineering The Chinese University of Hong Kong Hong Kong SAR China; ^11^ Xiangya School of Public Health Central South University Changsha China

**Keywords:** diabetes mellitus, machine learning, random survival analysis, risk score, uterine cancer

## Abstract

**Aim:**

This study aimed to develop predictive models and establish a risk scoring system to identify risk factors associated with survival in uterine cancer patients with type 2 diabetes (T2D) and estimate their survival probabilities.

**Methods:**

Data were collected from the Hong Kong Hospital Authority Data Collaboration Laboratory (HADCL) from 2000 to 2020. Cox proportional hazards regression, survival tree, LASSO Cox regression, boosting, and random survival forest (RSF) were utilized to develop predictive models for survival. Key risk factors were identified through Shapley Additive Explanations analysis, whereas the AutoScore‐Survival package facilitated the development of a risk scoring system.

**Results:**

This cohort study included 2047 uterine cancer patients with T2D. The average survival time was 100.82 (standard deviation: 72.75) months. The RSF model demonstrated the strongest predictive performance, achieving a time‐dependent area under the curve (AUC) of 0.823 and a *C*‐index of 0.90. A risk scoring system was created based on several criteria: age at cancer diagnosis, duration of T2D, creatinine levels, serum potassium level, low‐density lipoprotein cholesterol level (LDL‐C) level, body mass index (BMI), and triglycerides level. This scoring system classified 31.4% of patients as high‐risk, resulting in a 5‐year survival probability of 43.5%, about 1.7 times lower than that of the low‐risk group.

**Conclusion:**

This study leveraged machine learning to identify key survival predictors and develop a clinically interpretable risk scoring system for uterine cancer patients with T2D. Key predictors, including age at cancer diagnosis, duration of T2D, creatinine levels, serum potassium levels, LDL‐C levels, BMI, and triglycerides levels, effectively stratified survival risk. These findings demonstrate the potential of data‐driven models to enhance individualized prediction and inform targeted clinical management.

**Abbreviations**
AUC: area under the curve
AUC(*t*): time‐dependent AUC
BMI: body mass index
*C*‐index: concordance index
CoxPH: Cox proportional‐hazards
HADCL: Hospital Authority Data Collaboration Laboratory
HR: hazard ratio
iAUC: integrated AUC
ICD‐10: International Statistical Classification of Diseases and Related Health Problems, 10th Revision
ICD‐O‐3: International Classification of Diseases for Oncology, 3rd Edition
ICPC‐3: International Classification of Primary Care, 3rd Edition
KNN: k‐nearest neighbor
LDL‐C: low‐density lipoprotein cholesterol level
ML: machine learning
RAMP‐DM: Risk Assessment and Management Programme for Diabetes Mellitus
ROC: receiver operating characteristic
RSF: random survival forest
SD: standard deviation
SHAP: Shapley Additive Explanations
ST: survival tree
T2D: type 2 diabetes
UI: uncertainty interval

## 1 Background

Uterine cancer, often referred to as endometrial cancer, is the sixth most common cancer in women and ranks as the 15th most common cancer worldwide [1]. Globally, there were 435 041 incident cases (95% uncertainty interval [UI]: 245 710–272 470) and 91 640 deaths of uterine cancer (95% UI: 39 910–44 140) in 2019 [2]. Over the past 30 years, the age‐standardized incidence rate of uterine cancer has risen by 15.3%, whereas the mortality rate has decreased by 21.6% [2]. By 2044, incident cases of uterine cancer are projected to exceed 600 000 [2]. Type 2 diabetes (T2D) represents a parallel global health burden. In 2021, there were approximately 529 million (95% UI: 500–564) people living with diabetes worldwide, and the global age‐standardized total diabetes prevalence was 6.1% (5.8–6.5). By 2050, more than 1.31 billion (1.22–1.39) people are projected to have diabetes, most of whom will have T2D [3]. The coexistence of uterine cancer and T2D presents unique challenges, as T2D is associated with elevated risks of all‐cause and cancer‐specific deaths among uterine cancer survivors [4, 5].

Epidemiological studies consistently demonstrate a strong association between T2D and uterine cancer. A meta‐analysis of prospective cohort studies showed that diabetes was associated with around an 81% increased risk of uterine cancer [6]. Another later meta‐analysis reported an 89% increased risk of uterine cancer in women with diabetes compared to those without [7]. These risks persist even after adjusting for shared risk factors, such as obesity [8, 9]. The pathophysiological mechanisms underlying this association include insulin resistance, hyperinsulinemia, and chronic inflammation, which promote tumorigenesis [10, 11].

Some studies attributed the relationship between T2D and uterine cancer to their shared risk factors, such as obesity, physical inactivity, and aging [12]. Obesity, a shared risk factor for both conditions, further amplifies the risk [13, 14]. Other factors such as hypertension, cardiovascular disease, and reduced access to curative treatments also contribute to poorer outcomes in uterine cancer patients with T2D [15, 16]. Although some studies suggest no significant differences in treatment allocation by T2D status [17], evidence indicates that comorbidities in patients with T2D may complicate surgical or oncological treatment [15, 16]. Interestingly, metformin, a common antidiabetic medication, has shown promise in improving survival outcomes in patients with uterine cancer. Two meta‐analysis involving 1594 and 3923 women found that metformin use was associated with improved overall and progression‐free survival [18, 19]. However, the mechanistic basis for these benefits and their long‐term implications requires further investigation [6, 7, 20, 21].

Existing prognostic studies have explored the impact of different risk factors on the survival of uterine cancer, considering reproductive factors, physical inactivity, hypertension, and family history of uterine cancer (especially among close relatives) [22, 23, 24]. In a previous study conducted in Iowa, older women tend to have a higher risk of death, and alcohol drinking, parity, age at menarche, age at menopause, and education level are associated with the incidence of uterine cancer [4]. Despite this extensive body of research, there is a lack of studies focusing on risk factors associated with the prognosis of uterine cancer patients with T2D. The prognostic factors interplay between these two conditions remain poorly understood. This gap underscores the need for reliable and clinically actionable models to improve survival predictions and guide personalized care strategies for this vulnerable patient population [25].

Traditional statistical methods have limitations in analyzing high‐dimensional datasets that integrate diverse risk factors and outcomes. Machine‐learning (ML) techniques offer a promising solution by enabling the identification of complex patterns and relationships in large datasets [26]. This study aims to leverage ML techniques to develop predictive models for survival in uterine cancer patients with T2D. By identifying critical prognostic factors and constructing a validated risk score system, this research seeks to improve survival outcomes and enable more tailored treatment strategies. ML‐driven insights will help clinicians better understand the relevance of individual patient characteristics, optimize treatment decisions, and ultimately enhance care for uterine cancer patients with T2D.

## 2 Methods

### 2.1 Study Design and Data Source

This retrospective, territory‐wide cohort study utilized data from the Hong Kong Hospital Authority Data Collaboration Laboratory (HADCL) [27], which involved the Risk Assessment and Management Programme for Diabetes Mellitus (RAMP‐DM), with more than 90% of patients with T2D in Hong Kong receiving care through public healthcare institutions [28]. This program provides systematic risk stratification and detailed records on complications, disease control, medications, and lifestyle factors, offering invaluable data for this study.

### 2.2 Study Population and Data Collection

The study population comprised adult patients diagnosed with both uterine cancer and T2D between 2000 and 2020. Uterine cancer cases were identified using the International Classification of Diseases for Oncology, 3rd Edition (ICD‐O‐3) and the International Statistical Classification of Diseases and Related Health Problems, 10th Revision (ICD‐10, code C55) [29]. T2D was defined using ICD‐10 codes (5A11) or the International Classification of Primary Care, 3rd Edition (ICPC‐3, code TD72) [30, 31].

To ensure accuracy, only patients with uterine cancer listed as their “principal diagnosis” in the Diagnosis Progress dataset were included. Patients diagnosed with other cancers, type 1 diabetes, or gestational diabetes, as well as those lost to follow‐up, were excluded. The index date was defined as the date of uterine cancer diagnosis, with patients followed until death or the study endpoint (December 2020). Mortality data, including deaths occurring within or outside public hospitals, were sourced from the Hong Kong Death Registry. Other data included laboratory test results, medication details, procedures, and health behavior variables closest to the uterine cancer diagnosis date. These baseline measurements were used for analysis to ensure consistency and to reflect patients' physiological status at a clinically relevant stage. Variables with less than 90% completeness were excluded, and missing data for the remaining variables were imputed using the k‐nearest neighbor (KNN) algorithm. To standardize data, nonbinary variables were transformed into dummy variables, and continuous variables were categorized based on clinically relevant thresholds.

### 2.3 Statistical Methods and Modeling

Baseline patient characteristics were summarized using descriptive statistics. Cox proportional‐hazards (CoxPH) regression was employed to assess the relationship between individual variables and survival time, with Kaplan–Meier curves plotted for significant predictors to visualize their effects. A multivariable Cox regression model was then used to identify independent predictors of survival, adjusting for confounders and potential interactions. For survival prediction analysis, five ML algorithms were implemented: CoxPH model, penalized Cox model with LASSO penalty, CoxBoost, survival tree (ST), and random survival forest (RSF).

Data were split into 70% for training and 30% for testing. The characteristics for patients in the training set were contrasted with patients in the testing set, and the chi‐squared test or Fisher exact test was used to assess the validity of random distribution as appropriate. Hyper‐parameter optimization for the ML models was performed using a grid search strategy (details provided in the Supporting Information). Model performance was assessed using the concordance index (*C*‐index) and time‐dependent mean AUC, with scores ranging from 0.0 to 1.0, where higher values indicated better model fit. A 5‐fold cross‐validation was conducted twice to benchmark model performance. Missing data were imputed using KNN only in the training set for each fold, ensuring no data leakage between training and testing sets. To evaluate feature importance, Shapley Additive Explanations (SHAP) values were used to quantify each feature's impact on survival status and times in each model. This approach enhanced interpretability by linking feature importance to model predictions.

### 2.4 Risk Scores Development

An interpretable risk scoring system was developed using AutoScore‐Survival, an extension of the AutoScore framework tailored for time‐to‐event outcomes. AutoScore‐Survival integrates Cox regression modeling and ML to generate parsimonious survival models using right‐censored data [32]. Data were also split into training (70%) and testing (30%) sets, with the training set used for variable ranking, selection, and score generation, and the testing set reserved for independent validation of the resulting models. RSF analysis was conducted to rank variables based on nonlinear and heterogeneous effects. Parsimony plots and variable rankings were used to balance model accuracy and complexity. Continuous variables were converted into categorical variables based on predefined thresholds to better capture nonlinear effects. Scoring weights were derived from *β* coefficients in multivariable Cox regression, with the smallest coefficient serving as the reference.

Individual risk scores were calculated by summing the scores for selected variables, resulting in a total risk score ranging from 0 to 100. Higher scores corresponded to greater mortality risk. AutoScore‐Survival also allowed for customization of cutoff points based on clinical standards, ensuring alignment with professional norms and real‐world applicability. Receiver Operating Characteristic (ROC) analysis was conducted on the training set to evaluate overall model performance, with AUC values calculated. To assess predictive accuracy at specific time points, time‐dependent AUC (AUC(*t*)), integrated AUC (iAUC), and Harrell's *C*‐index were reported. Kaplan–Meier survival curves were plotted, and survival rates at key intervals (1, 3, and 5 years) were analyzed for risk groups defined by varying thresholds.

### 2.5 Ethical Approval

This study was approved by the Survey and Behavioral Research Ethics Committee of the Chinese University of Hong Kong (approval no. SBRE‐22‐0303A) and the joint CUHK and NTEC Clinical Research Ethics Committee (CREC) (2024.423). As the data utilized in this retrospective analysis were de‐identified and contained no personal details, the committee waived the requirement for informed consent.

## 3 Results

### 3.1 Patient Characteristics

The cohort included 2047 uterine cancer patients with T2D. Patient characteristics, laboratory results, and treatment details are summarized in Table S1. The mean (standard deviation, SD) age was 59.57 (10.42) years, and the mean (SD) BMI was 28.11 (5.24) kg/m^2^. At the time of data extraction, 1745 patients (85.25%) were alive, and 302 (14.75%) had died, with a mean (SD) survival time of 100.82 (72.75) months, and a mean duration between the diagnosis of T2D and the time of uterine cancer diagnosis (named “duration of T2D” thereafter) of 2.73 (3.43) years. A total of 1081 patients (52.81%) had a family history of diabetes. Most patients were non‐smokers (95.02%) and non‐drinkers (89.69%).

Regarding comorbidities, 1714 patients (83.73%) had central obesity, 121 (5.91%) had coronary heart disease, and 106 (5.18%) had a history of stroke. Laboratory test results revealed a mean cholesterol level of 4.54 mmol/L (SD: 0.96 mmol/L), mean HbA1c of 7.21 mmol/L (SD: 1.30 mmol/L), and mean fasting glucose of 7.56 mmol/L (SD: 2.16 mmol/L). In terms of treatment regimens, 199 patients (9.72%) were using insulin, 1719 patients (83.98%) were on antidiabetic medications, 1634 patients (79.82%) were prescribed antihypertensive drugs, and 1219 patients (59.55%) were receiving anti‐lipid drugs. There were no significant differences in patient characteristics between the training and test datasets.

### 3.2 Survival Analysis

**FIGURE 1:**
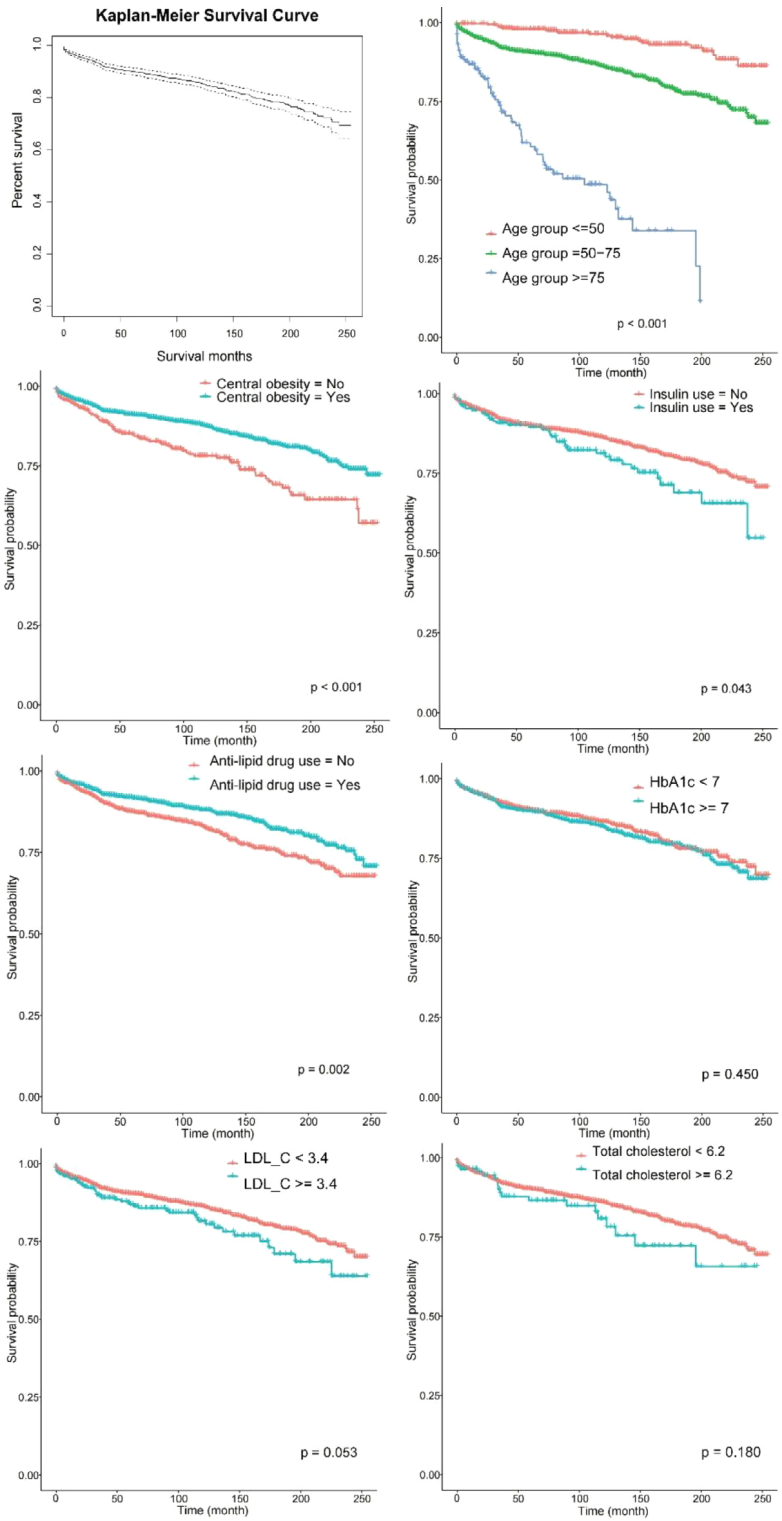
Kaplan–Meier curve plots for significant variables in univariable Cox regression. HbA1c, glycated hemoglobin; central obesity, waist circumference of men ≥ 90 cm or women ≥ 80 cm; LDL‐C, low‐density lipoprotein cholesterol level.

The survival functions estimated using Kaplan–Meier analysis are shown in Figure 1. Stratification by key clinical factors, including anti‐lipid drug use, central obesity, insulin use, and age group, revealed significant differences in survival outcomes. The median survival time for the cohort was 89.3 months. Results of the univariable and multivariable Cox regression analyses are summarized in Table S2. In the univariable analysis, seven variables were found to be significantly associated with a higher risk of mortality. These included age at cancer diagnosis (hazard ratio [HR]: 1.08, 95% confidence interval [CI]: 1.07–1.09, *p* < 0.001), duration of T2D (HR: 1.17, 95% CI: 1.14–1.20, *p* < 0.001), total cholesterol level (HR: 1.20, 95% CI: 1.08–1.33, *p* < 0.001), low‐density lipoprotein cholesterol level (LDL‐C; HR: 1.26, 95% CI: 1.12–1.41, *p* < 0.001), creatinine level (HR: 1.00, 95% CI: 1.00–1.00, *p* < 0.001), HbA1c (HR: 1.10, 95% CI: 1.02–1.19, *p* = 0.013), and insulin use (HR: 1.41, 95% CI: 1.01–1.98, *p* = 0.044). Conversely, two factors were significantly associated with a reduced risk of mortality: central obesity (HR: 0.57, 95% CI: 0.44–0.74, *p* < 0.001) and anti‐lipid drug use (HR: 0.69, 95% CI: 0.55–0.87, *p* = 0.002). In the multivariable analysis, four factors emerged as significant predictors of increased mortality risk: age at cancer diagnosis (HR: 1.07, 95% CI: 1.06–1.09, *p* < 0.001), duration of T2D (HR: 1.12, 95% CI: 1.09–1.16, *p* < 0.001), BMI (HR: 1.03, 95% CI: 1.01–1.06, *p* = 0.012), and creatinine level (HR: 1.00, 95% CI: 1.00–1.00, *p* = 0.025). Central obesity was the only variable found to be significantly associated with a decreased risk of mortality in the multivariable model (HR: 0.62, 95% CI: 0.46–0.83, *p* < 0.001).

### 3.3 Prediction Modeling

**FIGURE 2:**
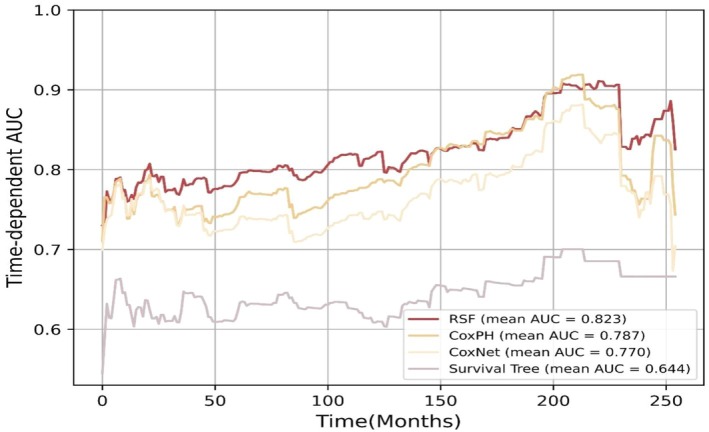
Time‐dependent area under the curve of the five machine learning algorithm. AUC, area under the curve; CoxPH, Cox proportional‐hazards; RSF, random survival forest.

**FIGURE 3:**
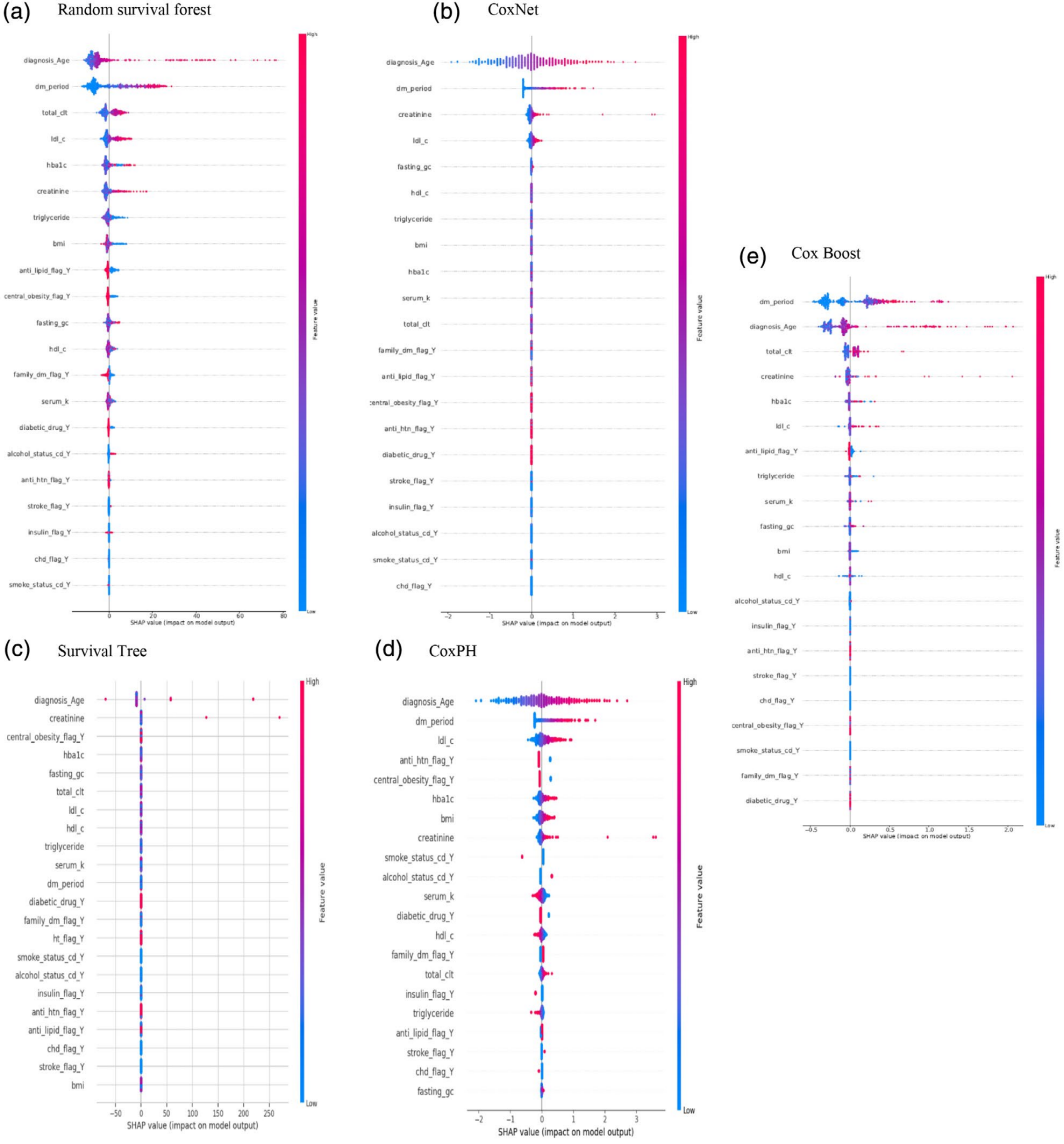
Shapley Additive Explanations (SHAP) feature importance visualization. (A) Random survival forest; (B) CoxNet; (C) survival tree; (D) CoxPH; (E) CoxBoost. Alcohol_status_cd_Y, patient with alcohol consumption; Anti_htn_flag_Y, patient receiving anti‐hypertensive drug; Anti_lipid_flag_Y, patient receiving anti‐lipid drugs; BMI, body mass index (kg/m^2^); Central_obesity_flag_Y, patient with the waist circumference of men ≥ 90cm or women ≥ 80cm; CHD_flag_Y, patient diagnosed with coronary heart disease; Diabetic_drug_Y, patient receiving antidiabetic drug; Diagnosis_age, the age of diagnosis with uterine cancer; DM_period, the duration between diagnosis of type 2 diabetes and the time of cancer diagnosis; Family_dm_flag_y, patient who had family history of diabetes; Fasting_gc, fasting glucose; HDL_c, high‐density lipoprotein cholesterol; Insulin_flag_Y, patient with insulin use; LDL‐C, low‐density lipoprotein cholesterol level; Smoke_status_cd_Y, smoker; Stroke_flag_Y, patient with history of stroke; Total_clt, total cholesterol level.

**FIGURE 4:**
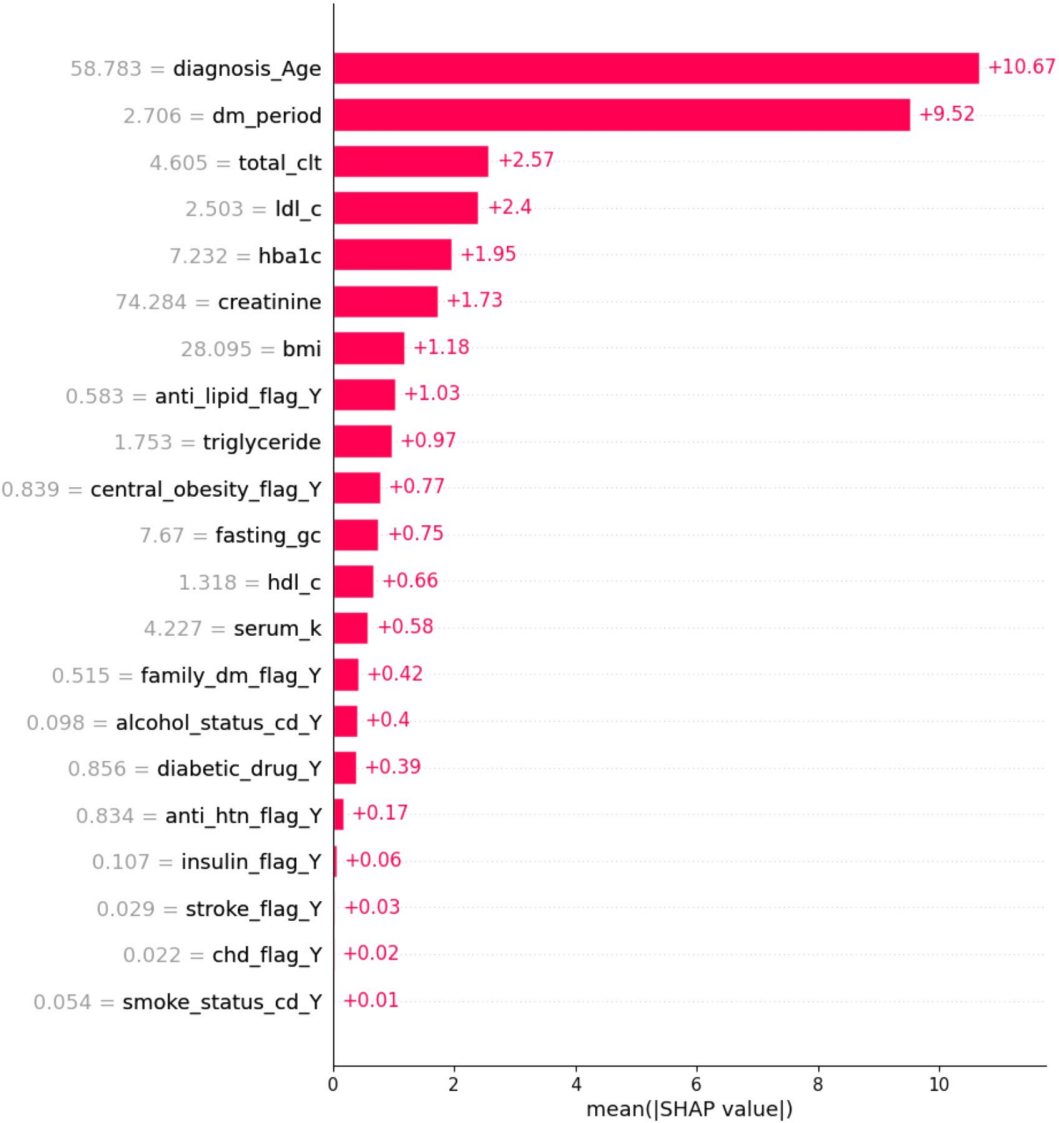
Shapley Additive Explanations (SHAP) values of the RSF model. Alcohol_status_cd_Y, patient with alcohol consumption; Anti_htn_flag_Y, patient receiving anti‐hypertensive drug; Anti_lipid_flag_Y, patient receiving anti‐lipid drugs; BMI, body mass index (kg/m^2^); Central_obesity_flag_Y, patient with the waist circumference of men ≥ 90cm or women ≥ 80cm; CHD_flag_Y, patient diagnosed with coronary heart disease; Diabetic_drug_Y, patient receiving antidiabetic drug; Diagnosis_age, the age of diagnosis with uterine cancer; DM_period, the duration between diagnosis of type 2 diabetes and the time of cancer diagnosis; Family_dm_flag_y, patient who had family history of diabetes; Fasting_gc, fasting glucose; HDL_c, high‐density lipoprotein cholesterol; Insulin_flag_Y, patient with insulin use; LDL‐C, low‐density lipoprotein cholesterol level; Smoke_status_cd_Y, smoker; Stroke_flag_Y, patient with history of stroke; Total_clt, total cholesterol level.

The performance of predictive models, as evaluated using the *C*‐index and time‐dependent AUC, is summarized in Table S3 and visualized in Figure 2. Among the models, the RSF model demonstrated superior performance, achieving the highest *C*‐index values of 0.90 for the training set and 0.73 for the test set, along with the highest mean time‐dependent AUC of 0.823. In contrast, the ST model exhibited the lowest performance, with a *C*‐index of 0.72 for the training set and 0.62 for the test set, and a mean time‐dependent AUC of 0.644. The importance of variables using SHAP summary plots was presented in Figure 3. Across all models, age at cancer diagnosis and duration of T2D were identified as the most influential factors, except in the ST model, where creatinine was ranked as the second most important factor. Figure 4 highlights the SHAP values for the best‐performing model (i.e., RSF), with the top five factors including age at cancer diagnosis (SHAP value: 10.67), duration of T2D (9.52), total cholesterol level (2.57), LDL‐C level (2.40), and HbA1c level (1.95).

### 3.4 AutoScore‐Survival Risk Score System

**FIGURE 5:**
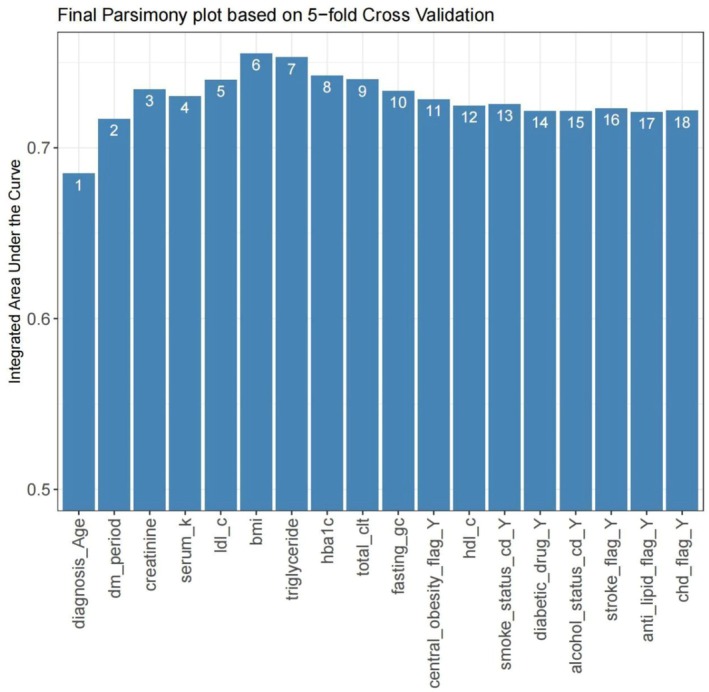
Model performance versus model complexity for AutoScore‐Survival system. Alcohol_status_cd_Y, patient with alcohol consumption; Anti_lipid_flag_Y, patient receiving anti‐lipid drugs; BMI, body mass index (kg/m^2^); Central_obesity_flag_Y, patient with the waist circumference of men ≥ 90cm or women ≥ 80cm; CHD_flag_Y, patient diagnosed with coronary heart disease; Diabetic_drug_Y, patient receiving antidiabetic drug; Diagnosis_Age, the age of diagnosis with uterine cancer; DM_period, the duration between diagnosis of type 2 diabetes and the time of cancer diagnosis; Fasting_gc, fasting glucose; HbA1c, glycated hemoglobin; HDL_C, high‐density lipoprotein cholesterol; LDL‐C, low‐density lipoprotein cholesterol; Smoke_status_cd_Y, smoker; Stroke_flag_Y, patient with history of stroke; Total_clt, total cholesterol level. AUC represents the model performance; the number in the bar shows the number of variables included in the model, representing the model complexity.

The parsimony plot (Figure 5) indicates that the AUC was approximately 0.68 when only the variable of age was considered and increased to 0.75 when incorporating the seven most significant variables identified from the RFS model. Table S4 outlines our risk score model, which is constructed from these seven factors: age at cancer diagnosis, duration of T2D, creatinine levels, serum potassium level, LDL‐C level, BMI, and triglycerides level. The model was predefined using quantiles (0, 0.05, 0.2, 0.8, 0.95, 1) for the transformation of continuous variables into categorical variables. Additionally, we established custom cutoff values for the tuned model as follows: age at cancer diagnosis was categorized into < 50, 50–60 (8), 60–70 (13), and ≥ 70 (31) years; duration of T2D was categorized into < 1 (0), 1–5 (19), and ≥ 5 (26) years; creatinine levels were categorized into < 48 (11), 48–55 (0), 55–80 (1), 80–116 (3), and ≥ 116 (7) mmol/L; serum potassium levels were categorized into < 3.5 (17), 3.5–3.9 (7), 3.9–4.6 (2), 4.6–5 (4), and ≥ 5 (0) mmol/L; LDL‐C was categorized into < 3.4 (0) and ≥ 3.4 (12) mmol/L; BMI was categorized into < 24 kg/m^2^ (0) and ≥ 24 kg/m^2^ (0); and triglycerides were categorized into < 1.7 (2) and ≥ 1.7 (0) mmol/L. Model performance is evaluated through the 1‐, 3‐, and 5‐year AUC(*t*) and *C*‐index, as illustrated in Table S5. The tuned model demonstrated superior performance, exhibiting a higher *C*‐index (0.784 vs. 0.769) and a greater 5‐year AUC(*t*) (0.807 vs. 0.799) compared to the predefined model.

**FIGURE 6:**
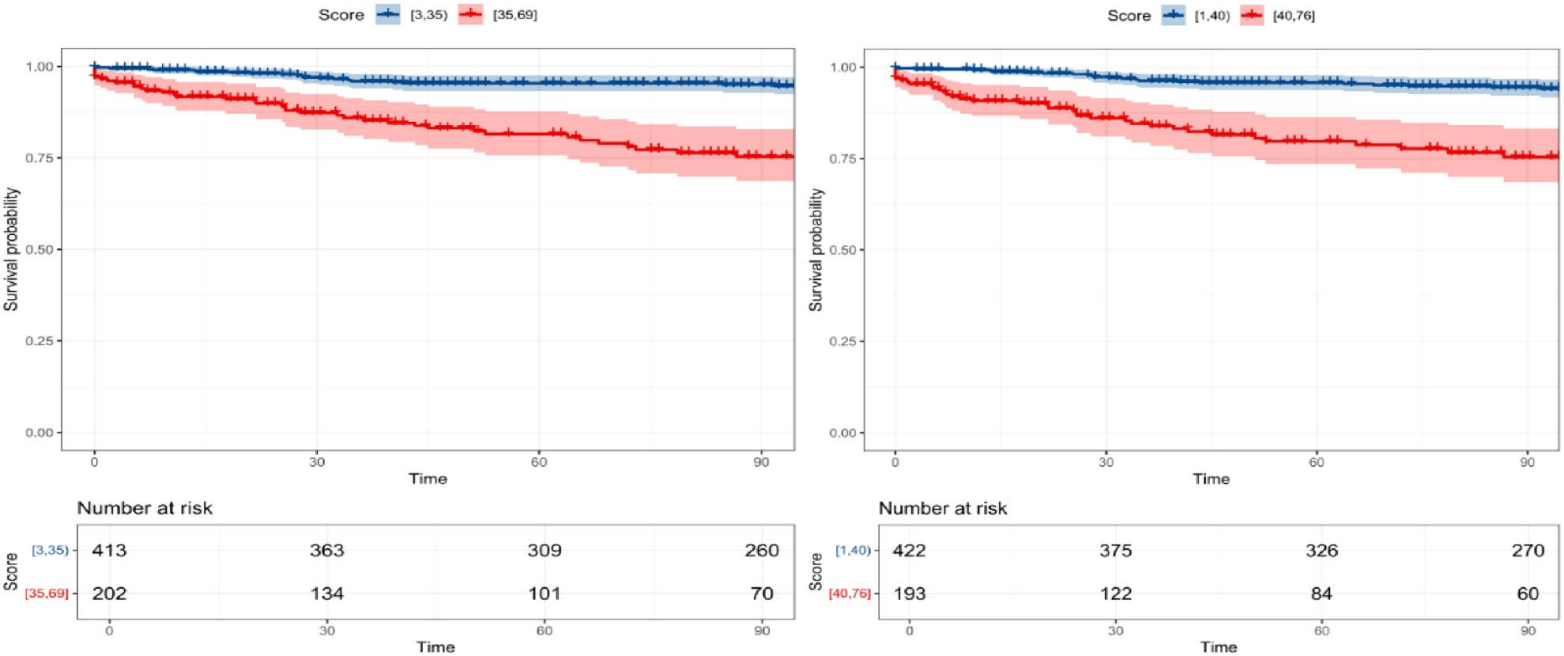
Kaplan–Meier survival curves under cutoff in predefined (left) and tuned (right) model.

Table S6 displays the survival probabilities at 1, 3, and 5 years for stratified risk groups under different models. In the predefined model, with a score cutoff of 35, 32.8% of patients were classified as high‐risk, with a 5‐year survival probability of 50.0%, approximately 1.5 times lower than that of the low‐risk group (74.8%). In the tuned model, with a score cutoff of 40, 31.4% of patients were classified as high‐risk, resulting in a 5‐year survival probability of 43.5%, about 1.7 times lower than that of the low‐risk group (62.5%). Figure 6 illustrates the Kaplan–Meier curve, demonstrating the stratification of the predefined model into score ranges of 3–34 and 35–69, with initial patient counts of 413 and 202, respectively, which decreased to 260 and 70 after 90 months. For the tuned model, scores were categorized into 1–39 and 40–76, with initial patient counts of 422 and 193, which subsequently decreased to 270 and 60 after 90 months in the corresponding lower and higher score groups.

## 4 Discussion

### 4.1 Summary of Findings

This population‐wide cohort study retrospectively assessed 2047 uterine cancer patients with T2D and successfully constructed a robust risk score system for the survival of patients using risk factors identified. In this study, we found that the RSF model outperformed all other ML algorithms, including CoxBoost, CoxPH, CoxNet, and ST models, and showed the best predictive performance for predicting the prognosis of uterine cancer patients with T2D, with the highest mean AUC and the best *C*‐index. We found age at cancer diagnosis, duration of T2D, BMI, and creatinine to be positively associated with the risk of mortality, whereas central obesity was negatively associated with the risk of mortality. The risk score system was developed based on seven factors, including age at cancer diagnosis, duration of T2D, creatinine level, serum potassium level, LDL‐C level, BMI, and triglyceride level. Notably, 31.4% of patients were categorized as high‐risk with scores exceeding 40, which corresponded to a 5‐year survival probability of 43.5%.

### 4.2 Possible Explanations and Comparisons With Previous Literature

Our findings underscore the significant role of various risk factors and the developed risk score system in predicting survival among uterine cancer patients with T2D. Specifically, the association of age at diagnosis with increased mortality risk corroborates findings from previous studies [4]. A longitudinal analysis of 41 836 women aged 55–69 years indicated that older age correlates with a heightened risk of death following an endometrial cancer diagnosis [4]. This relationship may be attributed to age‐related biological changes, such as compromised immunity and diminished cellular repair mechanisms, which could contribute to poorer clinical outcomes among older patients [33, 34, 35].

A longer duration of T2D has been associated with an increased risk of mortality. Prior research has similarly identified an association between prolonged diabetes duration and increased mortality risk, with a relative risk of 3.98 for women with diabetes duration exceeding 13 years compared to nondiabetic counterparts [4]. Additionally, our study identified a significant correlation between higher BMI and increased mortality risk among uterine cancer patients with T2D. Systematic reviews have similarly established that elevated BMI serves as a poor prognostic factor in uterine cancer, contributing to a substantial number of associated deaths [36, 37]. The underlying mechanisms may involve chronic obesity‐related ovarian hypogonadism, which, in conjunction with progesterone deficiency, fosters a cellular environment conducive to tumorigenesis. Excess adipose tissue linked to high BMI can worsen the prognosis of uterine cancer through hyperestrogenism; fat cells synthesize estrogen via aromatase activity, leading to elevated estrogen levels that may promote tumor growth and progression, thus contributing to a more aggressive cancer phenotype [22, 38]. Furthermore, high BMI is often associated with detrimental lifestyle habits, occupational stress, emotional disturbances, and comorbidities, which may further elucidate the observed relationship between elevated BMI and increased mortality among patients with cancer [36].

Serum potassium, triglycerides, and creatinine levels emerged as significant predictors of survival in this cohort. Elevated serum potassium levels have been implicated in various cancer types and may have prognostic implications, particularly when altered by immunotherapy [39]. Triglycerides, as markers of metabolic syndrome, were associated with worse survival outcomes, likely due to their role in supporting the energy demands of rapidly proliferating cancer cells. A previous prospective cohort study found that elevated triglyceride levels, indicative of metabolic syndrome, were associated with worse overall and disease‐free survival in endometrial cancer survivors, a finding that aligns with our study [40]. The mechanisms underlying this association may involve triglycerides serving as a source of energy and building blocks for rapidly growing cancer cells. High triglyceride levels can enhance the availability of fatty acids, which cancer cells utilize for energy production and membrane synthesis, thereby facilitating tumor growth and proliferation [41, 42]. Regarding creatinine levels, a case report and literature review suggested that elevated serum creatinine may result from targeted cancer therapies that inhibit tubular secretion [43]. Consequently, elevated creatinine levels may be associated with cancer or cancer‐related therapies, potentially increasing the risk of mortality in patients with uterine cancer through impaired kidney function [43, 44]. Further research is needed to evaluate the underlying mechanisms contributing to this association.

### 4.3 Strengths and Limitations

This study possesses several strengths. First, the large sample size of 2047 uterine cancer patients with T2D enhances the statistical power and generalizability of our findings. Second, the use of both univariable and multivariable Cox regression analyses highlighted significant risk factors, emphasizing the importance of comprehensive patient evaluation. Third, the integration of multiple ML algorithms facilitated the development of a robust predictive model for survival outcomes, demonstrating superior accuracy compared to traditional approaches. Last but not least, the practical and interpretable risk score system proposed in this study allows for straightforward application in clinical practice.

However, several limitations should be acknowledged. Notably, the study did not include variables that may potentially influence survival, such as tumor subtype, FIGO stage, or histologic subtype, treatment modalities, and adherence to treatment guidelines due to the unavailability of the data in the dataset [45]. Future research could extract this information using a large language model. Additionally, the reliance on data from the Hong Kong public healthcare system may limit the applicability of our findings to other healthcare contexts. For instance, the onset of the cancer might be much earlier than confirmation by diagnosis. Although internal validation was conducted using cross‐validation and a train‐test split, external validation across diverse populations was not performed due to data limitations. Such external validation is essential for assessing the model's robustness and applicability across different contexts. Moreover, the retrospective design limits causal inference, indicating that observed associations may not imply direct relationships. Further studies are needed to explore these variables and further investigate the underlying mechanisms.

### 4.4 Implications for Practice and Research

The developed risk score system offers a systematic method for stratifying patients based on individual risk profiles, enabling personalized medical strategies. Healthcare professionals can use this system to provide evidence‐based recommendations, such as lifestyle modifications for patients with elevated BMI, to improve clinical outcomes. As diagnostic and treatment practices continue to evolve, future updates using contemporary data will be essential to maintain the model's accuracy and applicability across changing patient populations. For research, the incorporation of ML algorithms in our predictive model underscores the potential of advanced analytical techniques in cancer research, suggesting avenues for future studies in various cancer types. Furthermore, the limitations identified in this study emphasize the necessity for ongoing research. Future studies should aim to incorporate more complete clinical data—including tumor characteristics and treatment‐related variables—to refine the risk score system and evaluate the added value of ML across broader uterine cancer cohorts. Moreover, the development of an open‐access web calculator could support clinical implementation and allow further testing of model generalizability across populations.

By performing ML prediction models, this population‐wide cohort study retrospectively assessing 2047 uterine cancer patients with T2D found that the RSF model outperformed all other ML algorithms, including CoxBoost, CoxPH, CoxNet, and ST models. Age at cancer diagnosis, duration of T2D, BMI, and creatinine were found to be positively associated with the risk of mortality and identified central obesity to be negatively associated with the risk of mortality. A risk score system was developed with 31.4% of patients categorized as high‐risk, which corresponded to a 5‐year survival probability of 43.5%. Further studies are expected to assess the applicability and validity of our risk score system in different settings and investigate the underlying mechanisms further.

## Author Contributions

**Claire Chenwen Zhong:** conceptualization, supervision, data curation, formal analysis, writing – original draft. **Junjie Huang:** conceptualization, supervision, writing – original draft. **Zehuan Yang:** writing – original draft. **Zhaojun Li:** formal analysis, data curation. **Yu Jiang:** data curation, formal analysis. **Jinqiu Yuan:** writing – review and editing. **Xiaodan Huang:** writing – review and editing. **Xiaofang Liu:** writing – review and editing. **Queran Lin:** writing – review and editing. **Han Wang:** writing – review and editing. **Jonathan Poon:** writing – review and editing. **Qi Dou:** writing – review and editing. **Irene Xin Yin Wu:** writing – review and editing. **Martin C. S. Wong:** conceptualization, supervision, writing – review and editing.

## Ethics Statement

Ethical approval was obtained from the Survey and Behavioral Research Ethics Committee, The Chinese University of Hong Kong (no. SBRE‐22‐0303A) and the Joint Chinese University of Hong Kong (CUHK) and New Territories East Cluster (NTEC) Clinical Research Ethics Committee (CREC) (2024.423).

## Consent

The authors have nothing to report.

## Conflicts of Interest

The authors declare no conflicts of interest.

## Supporting information


**Table S1:** Baseline characteristics of uterine cancer patients with type II diabetes.
**Table S2:** Univariable and multivariable Cox regression analysis.
**Table S3:** Concordance index (*C*‐index) of trained models.
**Table S4:** AutoScore‐Survival–derived scoring models.
**Table S5:** AUC(*t*) and *C*‐index of two AutoScore models implemented in train and test set in 1, 3, 5 years.
**Table S6:** Survival probabilities of patients with stratified risk scores at 1, 3, and 5 years in the two models.

## Data Availability

The data that support the findings of this study were requested from the Hong Kong Hospital Authority Data Collaboration Laboratory (HADCL) https://www3.ha.org.hk/data/DCL/Index/.
