# Supplementary material for: Machine Learning–Predictive Models for Survival in Uterine Cancer Patients With Type 2 Diabetes: A Territory‐Wide Cohort Study
Source: J Obstet Gynaecol Res. 2025 Sep 30;51(10):e70087. doi: 10.1111/jog.70087 (PMC12484716; doi:10.1111/jog.70087)
Supplement: Supplementary file 1 — Table S1: Baseline characteristics of uterine cancer patients with type II diabetes. Table S2: Univariable and multivariable Cox regression analysis. Table S3: Concordance index (C‐index) of trained models. Table S4: AutoScore‐Survival–derived scoring models. Table S5: AUC(t) and C‐index of two AutoScore models implemented in train and test set in 1, 3, 5 years. Table S6: Survival probabilities of patients with stratified risk scores at 1, 3, and 5 years in the two models. [file JOG-51-0-s001.docx]

**Supplementary Table**

Supplementary Table 1. Baseline characteristics of Uteri cancer Patients with Type II Diabetes

Supplementary Table 2. Univariable and multivariable Cox regression analysis

Supplementary Table 3 Concordance index (C-index) of trained models

Supplementary Table 4. AutoScore-Survival-derived scoring models

Supplementary Table 5. AUC(t) and C-index of two AutoScore models implemented in train and test set in 1, 3, 5 years

Supplementary Table 6. Survival probabilities of patients with stratified risk scores at 1, 3, and 5 years in the two models

**Supplementary Table 1. Baseline characteristics of Uteri cancer Patients with Type II Diabetes**

| Baseline characteristics | Train set  (N=1,432) | Test set  (N=615) | Total=2,047  N/mean(%/SD) | P value* |
| --- | --- | --- | --- | --- |
| Survival Time (months) | 97.97 ± 72.20 | 107.47 ± 73.64 | 100.82 ± 72.75 | 0.7172 |
| Survival Status  (N: alive; Y: dead) | N: 1,213 (84.71%) Y: 219 (15.29%) | N: 532 (86.50%) Y: 83 (13.50%) | N: 1,745 (85.25%) Y: 302 (14.75%) | 0.2835 |
| Age at cancer diagnosis (years) | 59.53 ± 10.22 | 59.67 ± 10.88 | 59.57 ± 10.42 | 0.7794 |
| Duration of T2D (years) | 2.74 ± 3.45 | 2.71 ± 3.39 | 2.73 ± 3.43 | 0.8827 |
| Family history of diabetes | Y: 764 (53.35%) N: 668 (46.65%) | Y: 317 (51.54%) N: 298 (48.46%) | Y: 1,081 (52.81%) N: 966 (47.19%) | 0.4534 |
| Smoker or not | N: 1,356 (94.69%) Y: 76 (5.31%) | N: 589 (95.77%) Y: 26 (4.23%) | N: 1,945 (95.02%) Y: 102 (4.98%) | 0.2831 |
| Alcohol consumption or not | N: 1,285 (89.73%) Y: 147 (10.27%) | N: 551 (89.59%) Y: 64 (10.41%) | N: 1,836 (89.69%) Y: 211 (10.31%) | 0.9235 |
| BMI (kg/m^2^) | 28.23 ± 5.16 | 27.81 ± 5.41 | 28.11 ± 5.24 | 0.1041 |
| Central_obesity | Y: 1,209 (84.43%) N: 223 (15.57%) | Y: 505 (82.11%) N: 110 (17.89%) | Y: 1,714 (83.73%) N: 333 (16.27%) | 0.2038 |
| CHD | N: 1,353 (94.48%) Y: 79 (5.52%) | N: 573 (93.17%) Y: 42 (6.83%) | N: 1,926 (94.09%) Y: 121 (5.91%) | 0.2677 |
| Stroke | N: 1,358 (94.83%) Y: 74 (5.17%) | N: 583 (94.80%) Y: 32 (5.20%) | N: 1,941 (94.82%) Y: 106 (5.18%) | 0.9734 |
| Total_cholesterol (mmol/L) | 4.57 ± 0.96 | 4.49 ± 0.97 | 4.54 ± 0.96 | 0.1057 |
| LDL_c (mmol/L) | 2.50 ± 0.85 | 2.41 ± 0.85 | 2.48 ± 0.85 | 0.2282 |
| HDL_c (mmol/L) | 1.30 ± 0.32 | 1.33 ± 0.35 | 1.31 ± 0.33 | 0.0902 |
| Triglyceride (mmol/L) | 1.71 ± 0.98 | 1.70 ± 1.19 | 1.71 ± 1.04 | 0.9692 |
| Serum potassium (mmol/L) | 4.23 ± 0.45 | 4.24 ± 0.45 | 4.23 ± 0.45 | 0.8731 |
| Creatinine (µmol/L) | 73.73 ± 48.82 | 73.73 ± 48.03 | 73.73 ± 48.57 | 0.9974 |
| HbA1c (%) | 7.21 ± 1.28 | 7.23 ± 1.32 | 7.21 ± 1.30 | 0.7399 |
| Fasting_glucose (mmol/L) | 7.55 ± 2.17 | 7.60 ± 2.14 | 7.56 ± 2.16 | 0.6587 |
| Insulin use | N: 1,292 (90.22%) Y: 140 (9.78%) | N: 556 (90.41%) Y: 59 (9.59%) | N: 1,848 (90.28%) Y: 199 (9.72%) | 0.8978 |
| Antidiabetic drug use | Y: 1,195 (83.45%) N: 237 (16.55%) | Y: 524 (85.20%)  N: 91 (14.80%) | Y: 1,719 (83.98%) N: 328 (16.02%) | 0.3130 |
| Antihypertensive drug use | Y: 1,144 (79.89%) N: 288 (20.11%) | Y: 490 (79.67%) N: 125 (20.33%) | Y: 1,634 (79.82%) N: 413 (20.18%) | 0.9124 |
| Anti-lipid drug use | Y: 839 (58.59%) N: 593 (41.41%) | Y: 380 (61.79%) N: 235 (38.21%) | Y: 1,219 (59.55%) N: 828 (40.45%) | 0.1744 |

T2D: Type 2 Diabetes; Duration of T2D: the duration between diagnosis of type 2 diabetes and the time of cancer diagnosis; BMI, Body Mass Index (kg/m^2^); Central_obesity, the waist circumference of men ≥ 90cm or women ≥ 80cm; CHD, indication on whether the patient is diagnosed with coronary heart disease; LDL_C, [low-density lipoprotein cholesterol](https://www.bing.com/ck/a?!&&p=e6c76973e8139b9bJmltdHM9MTcyMDY1NjAwMCZpZ3VpZD0xYTQ4OWMyMS1jMmNjLTYwMDMtMDVjYS04ZjhkYzNjNDYxNzkmaW5zaWQ9NTc4Ng&ptn=3&ver=2&hsh=3&fclid=1a489c21-c2cc-6003-05ca-8f8dc3c46179&psq=LDL-c&u=a1aHR0cHM6Ly9oZWFsdGhtYXR0ZXJzLmlvL3VuZGVyc3RhbmQtYmxvb2QtdGVzdC1yZXN1bHRzL2xkbC1j&ntb=1); HDL_C, high-density lipoprotein cholesterol; HbA1c, glycated hemoglobin; N: No, Y: Yes; F: female, M: male.

**Supplementary Table 2. Univariable and multivariable Cox regression analysis**

| Variables  (reference group) | Unadjusted HR  (95% CI) | P value* | Adjusted HR  (95% CI) | P value |
| --- | --- | --- | --- | --- |
| Age at cancer diagnosis | 1.08 (1.07, 1.09) | **<0.001** | 1.07 (1.06, 1.09) | **<0.001** |
| Duration of T2D (years) | 1.17 (1.14, 1.20) | **<0.001** | 1.12 (1.09, 1.16) | **<0.001** |
| Family history of diabetes (No) | 0.83 (0.66, 1.04) | 0.109 | 1.01 (0.79, 1.29) | 0.947 |
| Smoker or not (No) | 0.92 (0.54, 1.57) | 0.746 | 0.66 (0.38, 1.15) | 0.144 |
| Alcohol consumption or not (No) | 1.06 (0.73, 1.53) | 0.766 | 1.34 (0.92, 1.95) | 0.131 |
| BMI (kg/m^2^) | 0.98 (0.96, 1.00) | 0.117 | 1.03 (1.01, 1.06) | **0.012** |
| Central_obesity (No) | 0.57 (0.44, 0.74) | **<0.001** | 0.62 (0.46, 0.83) | **0.001** |
| Stroke (No) | 1.14 (0.72, 1.80) | 0.569 | 1.00 (0.63, 1.59) | 0.998 |
| Total cholesterol | 1.20 (1.08, 1.33) | **<0.001** | 0.90 (0.50, 1.64) | 0.742 |
| LDL_c | 1.26 (1.12, 1.41) | **<0.001** | 1.43 (0.78, 2.65) | 0.249 |
| HDL_c | 0.82 (0.58, 1.16) | 0.268 | 0.85 (0.42, 1.70) | 0.641 |
| Triglyceride | 1.04 (0.94, 1.15) | 0.448 | 1.01 (0.78, 1.29) | 0.959 |
| Serum potassium | 1.01 (0.78, 1.29) | 0.966 | 0.87 (0.68, 1.11) | 0.269 |
| Creatinine | 1.00 (1.00, 1.00) | **<0.001** | 1.00 (1.00, 1.00) | **0.025** |
| HbA1c | 1.10 (1.02, 1.19) | **0.013** | 1.07 (0.97, 1.19) | 0.164 |
| Fasting_glucose | 1.03 (0.98, 1.08) | 0.247 | 0.98 (0.93, 1.04) | 0.592 |
| Insulin use (No) | 1.41 (1.01, 1.98) | **0.044** | 0.95 (0.65, 1.40) | 0.801 |
| Antihypertensive drug use (No) | 1.06 (0.79, 1.43) | 0.697 | 0.75 (0.54, 1.04) | 0.081 |
| Anti-lipid drug use (No) | 0.69 (0.55, 0.87) | **0.002** | 0.97 (0.76, 1.24) | 0.806 |
| Antidiabetic drug use (No) | 0.90 (0.67, 1.21) | 0.488 | 0.83 (0.60, 1.15) | 0.265 |

T2D: Type 2 Diabetes; Duration of T2D: the duration between diagnosis of type 2 diabetes and the time of cancer diagnosis; BMI, Body Mass Index (kg/m^2^); Central_obesity, the waist circumference of men ≥ 90cm or women ≥ 80cm; CHD, indication on whether the patient is diagnosed with coronary heart disease; LDL_C, [low-density lipoprotein cholesterol](https://www.bing.com/ck/a?!&&p=e6c76973e8139b9bJmltdHM9MTcyMDY1NjAwMCZpZ3VpZD0xYTQ4OWMyMS1jMmNjLTYwMDMtMDVjYS04ZjhkYzNjNDYxNzkmaW5zaWQ9NTc4Ng&ptn=3&ver=2&hsh=3&fclid=1a489c21-c2cc-6003-05ca-8f8dc3c46179&psq=LDL-c&u=a1aHR0cHM6Ly9oZWFsdGhtYXR0ZXJzLmlvL3VuZGVyc3RhbmQtYmxvb2QtdGVzdC1yZXN1bHRzL2xkbC1j&ntb=1); HDL_C, high-density lipoprotein cholesterol; HbA1c, glycated hemoglobin; HR > 1 indicating an increased risk of the outcome (death).

*P value, calculated by Cox regression

**Supplementary Table 3. Concordance index (C-index) of trained models**

|  | Train set | Test set |
| --- | --- | --- |
| RSF | 0.90 | 0.73 |
| Coxboost | 0.85 | 0.73 |
| CoxPH | 0.80 | 0.72 |
| Coxnet | 0.78 | 0.70 |
| Survival Tree | 0.72 | 0.62 |

RSF, Random survival forest.

**Supplementary Table 4. AutoScore-Survival-derived scoring models**

|  | Predefined Model | | Tuned Model | |
| --- | --- | --- | --- | --- |
| Risk factor | Criteria | Score | Criteria | Score |
| Age at cancer diagnosis | <42 | 4 | <50 | 0 |
|  | [42,52) | 0 | [50,60) | 8 |
|  | [52,67) | 14 | [60,70) | 13 |
|  | [67,77) | 22 | >=70 | 31 |
|  | >=77 | 34 |  |  |
| Duration of T2D (years) | <5.2 | 0 | <1 | 0 |
|  | [5.2,9.95) | 10 | [1,5) | 19 |
|  | >=9.95 | 12 | >=5 | 26 |
| Creatinine (µmol/L) | <48 | 8 | <48 | 11 |
|  | [48,55) | 2 | [48,55) | 0 |
|  | [55,80) | 0 | [55,80) | 1 |
|  | [80,116) | 2 | [80,116) | 3 |
|  | >=116 | 7 | >=116 | 7 |
| Serum potassium (mmol/L) | <3.5 | 9 | <3.5 | 17 |
|  | [3.5,3.9) | 4 | [3.5,3.9) | 7 |
|  | [3.9,4.6) | 1 | [3.9,4.6) | 2 |
|  | [4.6,5) | 2 | [4.6,5) | 4 |
|  | >=5 | 0 | >=5 | 0 |
| LDL_c (mmol/L) | <1.38 | 0 | <3.4 | 0 |
|  | [1.38,1.82) | 2 | >=3.4 | 12 |
|  | [1.82,3.1) | 7 |  |  |
|  | [3.1,4.01) | 12 |  |  |
|  | >=4.01 | 19 |  |  |
| BMI (kg/m^2^) | <21.1 | 8 | <24 | 0 |
|  | [21.1,24.1) | 0 | >=24 | 0 |
|  | [24.1,32.1) | 2 |  |  |
|  | [32.1,37.4) | 6 |  |  |
|  | >=37.4 | 10 |  |  |
| Triglyceride (mmol/L) | <0.7 | 7 | <1.7 | 2 |
|  | [0.7,1) | 4 | >=1.7 | 0 |
|  | [1,2.2) | 0 |  |  |
|  | [2.2,3.5) | 0 |  |  |
|  | >=3.5 | 8 |  |  |

T2D: Type 2 Diabetes; Duration of T2D: the duration between diagnosis of type 2 diabetes and the time of cancer diagnosis; LDL_c, [low-density lipoprotein cholesterol](https://www.bing.com/ck/a?!&&p=e6c76973e8139b9bJmltdHM9MTcyMDY1NjAwMCZpZ3VpZD0xYTQ4OWMyMS1jMmNjLTYwMDMtMDVjYS04ZjhkYzNjNDYxNzkmaW5zaWQ9NTc4Ng&ptn=3&ver=2&hsh=3&fclid=1a489c21-c2cc-6003-05ca-8f8dc3c46179&psq=LDL-c&u=a1aHR0cHM6Ly9oZWFsdGhtYXR0ZXJzLmlvL3VuZGVyc3RhbmQtYmxvb2QtdGVzdC1yZXN1bHRzL2xkbC1j&ntb=1); BMI, Body Mass Index (kg/m^2^).

**Supplementary Table 5. AUC(t) and C-index of two AutoScore models implemented in train and test set in 1, 3, 5 years**

|  |  | Train set | | Test set | |
| --- | --- | --- | --- | --- | --- |
|  | Time_point | AUC_t | C_index | AUC_t (95% CI) | C_index (95% CI) |
| Predefined  Model | 1 year | 0.773 | 0.769 | 0.818 (0.725, 0.888) | 0.727 (0.673, 0.789) |
|  | 3 year | 0.799 |  | 0.700 (0.619, 0.799) |  |
|  | 5 year | 0.799 |  | 0.749 (0.671, 0.824) |  |
| Tuned  Model | 1 year | 0.809 | 0.784 | 0.866 (0.765, 0.921) | 0.759 (0.697, 0.807) |
|  | 3 year | 0.832 |  | 0.763 (0.675, 0.835) |  |
|  | 5 year | 0.807 |  | 0.795 (0.695, 0.875) |  |

AUC(t): Time-dependent area under the curve; C-index: Concordance index

**Supplementary Table 6. Survival probabilities of patients with stratified risk scores at 1, 3, and 5 years in the two models**

| **Risk Score Value** | | **Percent**  **of patients** | **Survival probability at 1 year (%)** | **Survival probability at 3 years (%)** | **Survival probability at 5 years (%)** |
| --- | --- | --- | --- | --- | --- |
| Predefined  Model | <35 | 67.2% | 95.9% | 86.2% | 74.8% |
|  | ≥35 | 32.8% | 80.2% | 61.4% | 50.0% |
| Tuned  Model | <40 | 68.6% | 96.0% | 87.2% | 77.3% |
|  | ≥40 | 31.4% | 79.3% | 58.0% | 43.5% |
